# Severe eosinophilic asthma in Chinese C‐BIOPRED asthma cohort

**DOI:** 10.1002/ctm2.710

**Published:** 2022-02-20

**Authors:** Qingling Zhang, Xiuhua Fu, Changzheng Wang, Huahao Shen, Lei Zhu, Guochao Shi, Zhongmin Qiu, Zhongguang Wen, Wei Gu, Wei Luo, Lina Zhao, Yunqin Chen, Sam Lim, Chang Xiao, Jian Kang, Yunhui Zhang, Mao Huang, Jinfu Xu, Kewu Huang, Qiang Li, Xiangyan Zhang, Jianping Zhao, Xiaoxia Liu, Shenghua Sun, Huaping Tang, Bei He, Shaoxi Cai, Ping Chen, Chunhua Wei, Guangfa Wang, Ping Chen, Lixin Xie, Jiangtao Lin, Yuling Tang, Zhihai Han, Kian Fan Chung, Nanshan Zhong, the C‐BIOPRED consortium

**Affiliations:** ^1^ Department of pulmonary and Critical Care Medicine, Guangzhou Institute of Respiratory Health, National Clinical Research Center for Respiratory Disease, National Center for Respiratory Medicine, State Key Laboratory of Respiratory Diseases The First Affiliated Hospital of Guangzhou Medical University Guangzhou China; ^2^ Department of Respiratory Medicine The Affiliated Hospital of Inner Mongolia Medical University Huhhot China; ^3^ Department of Respiratory Medicine, Xinqiao Hospital Army Military Medical University Chongqing China; ^4^ Department of Respiratory Medicine The Second Affiliated Hospital of Zhejiang University School of Medicine Hangzhou China; ^5^ Department of Respiratory Medicine Zhongshan Hospital Shanghai China; ^6^ Department of Respiratory Medicine, Ruijin Hospital Shanghai Jiaotong University School of Medicine Shanghai China; ^7^ Department of Respiratory Medicine Shanghai Tongji Hospital Shanghai China; ^8^ Department of Respiratory Medicine The First Affiliated Hospital of PLA General Hospital Beijing China; ^9^ Department of Respiratory, Nanjing First Hospital Nanjing Medical University Nanjing China; ^10^ AstraZeneca Shanghai China; ^11^ National Heart and Lung Institute Imperial College London, & Royal Brompton & Harefield NHS Foundation Trust London SW3 UK; ^12^ Department of Respiratory Medicine The First Hospital of China Medical University Shenyang China; ^13^ Department of Respiratory Medicine The First People's Hospital of Yunnan Province Kunming China; ^14^ Department of Respiratory Medicine Jiangsu Province Hospital Nanjing China; ^15^ Department of Respiratory Medicine Shanghai Pulmonary Hospital Shanghai China; ^16^ Department of Respiratory Medicine Beijing Chao‐Yang Hospital Beijing China; ^17^ Department of Respiratory Medicine Shanghai General Hospital Shanghai China; ^18^ Department of Respiratory Medicine Guizhou Province People's Hospital Guiyang China; ^19^ Department of Respiratory Medicine, Tongji Hospital, Tongji Medical College Huazhong University of Science and Technology Wuhan China; ^20^ Department of Respiratory Medicine, Beijing Friendship Hospital Capital Medical University Beijing China; ^21^ Department of Respiratory Medicine The Third Xiangya Hospital of Central South University Changsha China; ^22^ Department of Respiratory Medicine Qingdao Municipal Hospital Qingdao China; ^23^ Department of Respiratory Medicine Peking University Third Hospital Beijing China; ^24^ Department of Respiratory Medicine Nanfang Hospital of Southern Medical University Guangzhou China; ^25^ Department of Respiratory Medicine The General Hospital of Shenyang Military Shenyang China; ^26^ Department of Respiratory Medicine Weifang Asthma hospital Weifang China; ^27^ Department of Respiratory Medicine Peking University First Hospital Beijing China; ^28^ Department of Respiratory Medicine The Second Xiangya Hospital of Central South University Changsha China; ^29^ College of Pulmonary and Critical Care Medicine Chinese PLA General Hospital Beijing China; ^30^ Department of Respiratory Medicine China‐Japan Friendship Hospital Beijing China; ^31^ Department of Respiratory Medicine The First Hospital of Changsha Changsha China; ^32^ Department of Respiratory Medicine Navy General Hospital Beijing China

**Keywords:** blood eosinophil count, cigarette smoking, exacerbations, fractional exhaled nitric oxide, severe asthma

Dear Editor,

In China, asthma affects 45.7 million adults with a prevalence of 4.2%,^1^ while that of severe asthma ranges from 3.4% to 8.3% among asthmatic patients.^2^, ^3^, ^4^ There is scarce information on the characteristics and biomarker expression, and the different phenotypes of severe asthma. The C‐BIOPRED consortium recruited patients with severe asthma from 33 university hospitals in 15 provinces in China.^5^ These patients were receiving treatment with medium to high doses of inhaled corticosteroids and long‐acting β_2_‐agonists and experienced uncontrolled asthma, defined by the experience of two or more asthma exacerbations, requiring daily oral corticosteroids (OCS), and/or other asthma medication. The participants were all adults either with severe non‐smoking asthma (NSA; *n* *= *342), current and former smokers with severe asthma with a smoking history of >5 pack‐years (SSA; *n* *= *110), patients with mild to moderate asthma with controlled or partially controlled symptoms (MMA; *n* *= *93) and healthy non‐smoking controls (HC; *n* *= *100) (Figure S2; Table 1). The NSA and SSA groups had more symptomatic asthma as measured by the Asthma Control Questionnaire (ACQ) and Asthma Quality of Life Questionnaire (AQLQ) (Table S2) and they also reported more exacerbations in the previous year than MMA (Table 1). Measurement of forced expiratory volume in the first second (FEV_1_, litters or % predicted) and the ratio of FEV1 to forced vital capacity (FVC) (litters or % predicted), both measures of airflow obstruction were lower in severe asthma patients than in the MMA and HC groups (*p < *.001) (Table 1). The quality of life may be affected by airflow obstruction and exacerbations are supported by the significant correlations between AQLQ scores and FEV_1_ (L) (*r* *= *0.09; *p* < .05) and exacerbations in the previous year (*r* = −0.11; *p = *0064) (Figure S3).

**TABLE 1:** Demographic characteristics of C‐BIOPRED

**Characteristic**	**Severe nonsmoking asthma (*n* = 342)**	**Smokers and ex‐smokers with severe asthma (*n* = 110)**	**Mild/moderate nonsmoking asthma (*n* = 93)**	**Healthy nonsmoking controls (*n* = 100)**	***p*‐Value**
Gender					
Male	101 (29.5)	106 (96.4)	43 (46.2)	36 (36.0)	<.001
Female	241 (70.5)	4 (3.6)	50 (53.8)	64 (64.0)	
Age (years)	53.39 ± 11.45	57.31 ± 9.46	48.65 ± 12.07	33.18 ± 14.20	<.001
Age of diagnosis (years)	40.08 ± 16.42	44.46 ± 17.36	35.76 ± 16.07	NA	<.001
BMI (kg/m^2^)	24.31 ± 3.65	25.05 ± 3.25	24.21 ± 3.08	22.38 ± 2.81	<.001
BMI Normal (< 28)	293 (85.7)	90 (81.8)	84 (90.3)	98 (98.0)	<.001
BMI Obesity (> = 28)	49 (14.3)	20 (18.2)	9 (9.7)	2 (2.0)	
Smoking (Pack‐years)	NA	28.71 (22.76)	NA	NA	NA
Current Smoker	NA	44 (40.0)	NA	NA	NA
Ex‐smoker	NA	66 (60.0)	NA	NA	NA
pre‐BD FVC, L	2.57 ± 0.76	3.11 ± 0.80	3.35 ± 0.94	3.81 ± 0.97	<.001
pre‐BD FVC % pred, L	83.43 ± 17.38	80.76 ± 16.54	97.21 ± 17.54	101.50 ± 12.35	<.001
pre‐BD FEV_1_, L	1.55 ± 0.58	1.79 ± 0.71	2.21 ± 0.81	3.20 ± 0.79	<.001
pre‐BD FEV_1_% pred	64.79 ± 21.16	60.87 ± 21.22	81.69 ± 23.13	106.01 ± 11.44	<.001
pre‐BD FEV_1_/FVC, %	60.17 ± 12.29	56.72 ± 12.43	65.38 ± 11.65	84.19 ± 5.83	<.001
Post‐BD, n	329	108	80		
Post‐BD FEV1, L	1.82 ± 0.60	2.07 ± 0.72	2.45 ± 0.78	NA	<.001
Post‐BD FEV1% pred	76.02 ± 21.16	70.47 ± 21.93	91.03 ± 21.79	NA	<.001
Post‐BD FEV1 (% increase)	21.60 ± 15.45	17.88 ± 14.73	14.74±10.56	NA	<.001
Exacerbations in prior year	1.50 ± 1.80	1.36 ± 1.60	0.33 ± 0.54	NA	<.001
Exacerbation in the previous year, n(%)					
Yes	113 (33.0)	36(32.7)	64 (68.8)	NA	<.001
No	219 (64.0)	72 (65.5)	27 (29.0)	NA	
Healthcare resource utilization, n(%)					
Yes	64 (18.7)	14 (12.7)	5 (5.4)	NA	<.001
No	278 (81.3)	96 (87.3)	88 (94.6)	100 (100.0)	

Abbreviations: BD, bronchodilator; BMI, body mass index; FEV1, forced expiratory volume in one second; FVC, forced vital capacity; Max, maximum; Min, Minimum, N, number of subjects in the cohort, n, Number of subjects included in the analysis; NA, Not applicable; SD, Standard deviation.

*Note*: Data are shown as mean ± standard deviation, unless as n (%). The subject level data used for FVC, pre‐BD FEV1, pre‐BD FEV1% pred, pre‐BD FEV1/FVC ratio, % analysis follows the algorithm below: If baseline data are available, then baseline data are used. Otherwise, if screening data are available, then screening data are used.

The proportion of patients who had nasal polyps and/or nasal polypectomy and gastro‐oesophageal reflux disease in severe asthma was higher than that in the MMA and HC (Table S3). Atopy defined by at least one positive specific IgE level and total serum IgE was higher in the three asthma groups than among HCs (*p* < .001) (Table 2). Regarding asthma treatments, the NSA and SSA groups, 9.94% of NSA and 12.73% of SSA received daily OCS, and those in the MMA group received none (Table S1). Therefore, severe asthma patients have more symptoms, more exacerbations, more airflow obstruction, and more nasal polyps even if they are on high‐dose asthma medication regimens, including oral corticosteroids.

**TABLE 2:** Biomarkers in blood and exhaled air

	**Severe nonsmoking asthma(*n* = 342)**	**Smokers and ex‐smokers with severe asthma(*n* = 110)**	**Mild/moderate nonsmoking asthma(*n* = 93)**	**Healthy nonsmoking controls (*n* = 100)**	***p*‐Value**
Subjects with FeNO, n	320	103	75	86	
FeNO(ppb)	31.00 (20.00, 54.00)	27.00 (18.00, 59.50)	28.00 (19.00, 47.00)	15.00 (12.00, 19.75)	<.001
Subjects with blood results, n	341	107	93	100	
Neutrophil count(10^∧^9/L)	3.72 (2.91, 4.61)	4.10 (3.30, 4.98)	3.78 (2.91, 4.74)	3.45 (2.86, 4.34)	0.004
Eosinophil count(10^∧^9/L)	0.24 (0.11, 0.45)	0.24 (0.13, 0.46)	0.21 (0.13, 0.30)	0.08 (0.05, 0.13)	<.001
Neutrophil (%)	57.40 (51.80, 64.04)	61.00 (53.15, 66.65)	59.40 (53.60, 65.50)	58.70 (54.35, 62.75)	0.066
Eosinophil (%)	3.60 (1.90, 7.50)	3.50 (2.05, 6.45)	3.50 (2.00, 5.00)	1.20 (0.80, 2.22)	<.001
Subjects with IgE and ECP results, n	281	90	77	73	
ECP^*^(μg/L)	7.29 (4.00, 13.70)	7.71 (4.36, 15.13)	7.38 (3.73, 12.50)	3.37 (2.35, 5.54)	<.001
IgE Total (KU/L)	161.00 (58.50, 395.00)	232.00 (97.38, 534.50)	171.00 (68.00, 393.00)	32.40 (18.50, 94.30)	0.001
Atopy^*^					
above normal	137 (48.8)	49 (54.4)	48 (62.3)	19 (26.0)	<.001
normal	144 (51.2)	41 (45.6)	29 (37.7)	54 (74.0)	

Data are presented as median values (interquartile range) for continuous variables and as n (%) for categorical variables.

Abbreviations: ECP, eosinophil cationic protein; FeNO, fractional level of nitric oxide in exhaled breath; HX2, house dust mix; MX2, mold mix; TX4, tree pollen mix; WX5, weed pollen mix; FX5, food allergens.

Specific IgE (HX2, FX5, MX2, TX4, and WX5 Phadiatop) <0.35 Ku/L is considered non‐atopic.

*Note*: The Kruskal‐Wallis test was here used for continuous data and Fisher's exact test for discrete data.

*Note*: Atopy (above normal) means the subjects have at least one allergen above normal (> .35).

To understand the role of inflammatory factors in severe asthma, we measured blood eosinophil and neutrophil counts (BEC and BNC) and fractional level of nitric oxide in exhaled breath (FeNO), and the level of granulocytes in sputum (SEC and SNC) collected after inhalation of hypertonic saline solutions in a smaller group (260 out of 545 asthma participants). Levels of BEC, BNC, and serum eosinophil cationic protein (ECP), a product released from activated eosinophils, were higher in the NSA, SSA, and MMA than in the HC (Table 2). FeNO levels were also higher in the three asthma groups (Table 2). BEC (%) was correlated with FeNO (*r* *= *0.37; *p* < .001) (Figure S4), as were sputum eosinophils (%) (*r* *= *0.38; *p* < .0001) (Figure 1). Median SEC of NSA and SSA were 11.6% and 8.1%, respectively, which were higher than those of MMA (5.1%) and HC (0.9%) (*p* < .0001) (Table S4; Figure S5). There was no difference in SNC among the four groups. Using BEC ≥ 300/μl as a marker of Type‐2 inflammation, 38.4% of severe asthma can be categorized as severe eosinophilic asthma, while this would be 76.8% using a cut‐off for SEC (%) ≥ 2.5%. There was a correlation between SEC (%) and the number of exacerbations in the previous year (*r* *= *0.24; *p = *0.005), AQLQ score (*r* *= *−0.18; *p = *0.012), and ACQ5 score (*r* *= *0.17; *p = *.015) (Figure 1). SNC (%) was correlated with that of BNC (%) (*r* *= *0.26; *p *0.0002). In NSA and SSA groups, SNC (%) was negatively correlated with FEV_1_ (% predicted) (r* = *−0.28; *p *< .001) and FEV_1_/FVC ratio (*r* *= *−0.27; *p* < .001), but there was no correlation of SEC (%) with these parameters (Figure 1).

**FIGURE 1:**
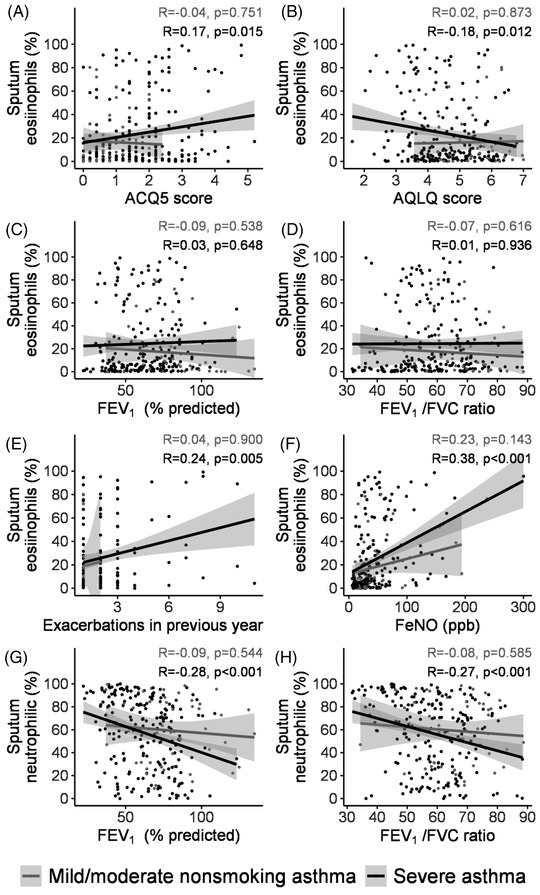
Correlations between sputum eosinophils and neutrophil (%), and ACQ‐5 score, AQLQ score, FEV_1_ (% predicted), FEV_1_/FVC ratio, exacerbations in the previous year, and FeNO for severe asthma and mild‐moderate asthma. The correlation coefficients (R) and *p*‐values are indicated

At the one‐year review in the NSA and SSA, there were no changes in airflow obstruction or the biomarkers of inflammation (BEC, BNC, and FeNO) indicating biostability of the phenotype. However, improvement in symptoms and asthma control was observed in NSA (Table 3).

**TABLE 3:** Data for baseline and longitudinal visits

	**Severe nonsmoking asthma (*n* = 205)**		**Smokers and ex‐smokers with severe asthma (*n* = 58)**		**SNA**	**SSA**
	**Baseline**	**Longitudinal**	**Baseline**	**Longitudinal**	***p*‐Value**	***p*Value**
Longitudinal visit subjects with pulmonary test, n	205	204	58	58		
pre‐BD FVC, L (Mean (SD))	2.61 (0.75)	2.64 (0.74)	3.04 (0.70)	3.15 (0.76)	0.640	0.501
pre‐BD FEV1, L (Mean (SD))	1.56 (0.56)	1.61 (0.54)	1.71 (0.60)	1.81 (0.72)	0.208	0.545
pre‐BD FEV1% predicted (Mean (SD))	65.62 (21.54)	68.85 (21.09)	58.38 (18.89)	62.21 (22.39)	0.093	0.436
pre‐BD FEV1/FVC, %(Mean(SD))	59.50 (12.34)	60.92 (12.20)	55.74 (12.30)	56.23 (13.13)	0.222	0.804
Longitudinal visit subjects with bronchodilator reversibility test, n	190	190	56	56		
FEV1 reversibility (L), median(IQR)	0.28 (0.22, 0.38)	0.18 (0.09, 0.27)	0.27 (0.18, 0.42)	0.23 (0.16, 0.37)	<.001	0.322
FEV1 reversibility (%), median (IQR)	20.74 (13.66, 31.11)	12.12(5.91, 20.75)	15.98 (8.78, 31.10)	14.90 (8.31, 22.28)	<.001	0.480
Healthcare resource utilization,n(%)	205	205	58	58		
Yes	43 (21.0)	64 (31.2)	7 (12.1)	16 (27.6)	0.024	0.061
No	162 (79.0)	141 (68.8)	51 (87.9)	42 (72.4)		
Subjects with questionnaire, n	205	205	57	57		
ACQ						
ACQ5	1.65 (1.03)	1	1.81 (0.98)	1.56 (1.07)	0.012	0.158
ACQ7	1.85 (0.86)	1.65 (0.82)	2.08 (0.85)	1.84 (0.94)	0.017	0.121
AQLQ						
AQLQ	4.54 (1.02)	4.83 (1.07)	4.71 (1.06)	4.91 (1.02)	0.005	0.296
Symptoms	4.76 (1.07)	5.06 (1.06)	4.78 (1.06)	5.09 (1.01)	0.004	0.129
Activity limitation	4.46 (1.02)	4.76 (1.09)	4.67 (1.21)	4.87 (1.16)	0.004	0.392
Emotional	4.44 (1.37)	4.75 (1.35)	4.69 (1.33)	4.79 (1.48)	0.018	0.716
Environmental	4.19 (1.46)	4.40 (1.50)	4.60 (1.46)	4.62 (1.50)	0.106	0.925
ESS	6.87 (4.2)	6.66 (4.4)	7.46 (4.42)	7.58 (4.73)	0.393	0.889
MARS	21.44 (2.92)	21.45 (3.33)	21.77 (2.8)	21.84 (2.69)	0.599	0.961
Subjects with FeNO, n	192	194	54	54		
FeNO(ppb)	29.50 [20.00, 52.00]	28.00 [18.25, 48.00]	26.50 [18.00, 63.50]	29.00 [16.25,48.50]	0.758	0.775
Subjects with blood results, n	205	197	58	57		
Neutrophil count(10^∧^9/L)	3.60 [3.00, 4.50]	3.54 [2.85, 4.59]	3.90 [3.27,4.95]	3.89 [2.90,5.21]	0.580	0.775
Eosinophil count(10^∧^9/L)	0.23 [0.11, 0.40]	0.23 [0.11, 0.44]	0.26 [0.13,0.40]	0.20 [0.12,0.42]	0.501	0.745
Neutrophil (%)	57.70 [52.20, 64.20]	58.90 [52.30, 64.70]	60.85 [52.85, 66.68]	59.10 [50.40, 67.00]	0.703	0.576
Eosinophil (%)	3.60 [1.90, 6.50]	4.20 [1.90, 7.00]	3.60 [2.18, 6.37]	3.30 [1.80, 6.00]	0.320	0.724
Subjects with IgE and ECP results, n	169	162	47	50		
ECP (μg/L)	7.77 [4.03, 16.20]	7.05 [4.10, 13.73]	6.98 [4.55, 14.95]	6.99 [4.30, 14.95]	0.389	0.745
IgE Total (Ku/L)	165.00 [57.80, 407.00]	168.50 [62.98, 378.75]	320.00 [128.50, 909.00]	321.00 [94.57, 1189.00]	0.760	0.940
Atopy*						
above normal	88 (52.07)	89 (54.94)	29 (61.70)	24 (48.00)	0.659	0.222
normal	81 (47.93)	73 (45.06)	18 (38.30)	26 (52.00)		

Data are presented as mean (standard deviation) or median (interquartile range) for continuous variables, n (%) for categorical variables.

*Note*: Using all severe patients without missing value.

Abbreviations: FEV1, forced expiratory volume in one second; FVC, forced vital capacity; BD. bronchodilator.

*Note*: *P*‐values are based on the comparison between baseline and longitudinal values. The Kruskal‐Wallis test was used for continuous data and the Fisher exact test for discrete data.

* Atopy (above normal) means the subjects have at least one above normal (>0.35) records for HX2, FX5, MX2, TX4, WX5, and Phadiatop.

This C‐BIOPRED study of patients with severe asthma in China uniquely defines a population with the most severe disease characterized by poorer asthma control, frequent exacerbations, and chronic airflow obstruction despite taking maximal amounts of asthma medication in terms of oral corticosteroid therapy, with ≈10% needing oral corticosteroid therapy which is associated with systemic side‐effects. The use of biomarkers in severe asthma to define those with Type‐2 inflammation that is associated with eosinophilic inflammation indicates that SEC is a better biomarker than BEC or FeNO in that respect^6^ because it could distinguish MMA from NSA and SSA. Thus, using SEC, a very high proportion of patients with severe asthma of up to 76.8% would have severe eosinophilic asthma, which is higher than in other severe asthma cohorts^7^, ^8^, ^9^ of Caucasian populations. This has implications for future therapies because of the current availability of biologic therapies such as anti‐IgE, anti‐IL5, anti‐IL‐5Rα, and anti‐IL4Rα monoclonal antibodies for Type‐2 severe asthma.^10^

These findings provide insight into the role of some of the factors driving various traits of severe asthma. Thus, the degree of airflow obstruction and the exacerbation rate may determine the level of deterioration while the eosinophilic inflammation may underlie the rate of exacerbations. Indeed, the amelioration in severe asthma provided by the anti‐IL5 or anti‐IL5Rα antibodies that suppress eosinophil inflammation led to a reduction in exacerbation rates.^10^ However, the negative correlation between the sputum neutrophil count and FEV_1_ indicated a possible role of neutrophils in determining airflow obstruction. Since severe asthma has different molecular phenotypes other than Type‐2,^11^ further exploration of molecular phenotypes in this Chinese cohort may facilitate precision medicine and allow further dissection of these multiple molecular pathways.^12^

## CONFLICT OF INTEREST

The authors declare no conflict of interest.

## FUNDING

This study was funded by Astra‐Zeneca, China.

## Supporting information

Supporting Information

## References

[1] Huang K , Yang T , Xu J , et al. Prevalence, risk factors, and management of asthma in China: a national cross‐sectional study. Lancet. 2019;394(10196):407‐418. 31230828 10.1016/S0140-6736(19)31147-X

[2] Su N , Lin JT , Wang WY , et al. A cross‐section study of severe asthma in eight provinces of China. Zhonghua Nei Ke Za Zhi. 2016;55(12):917‐921. 27916044 10.3760/cma.j.issn.0578-1426.2016.12.002

[3] Wang WY , Lin JT , Zhou X , et al. A survey on clinical characteristics and risk factors of severe asthma in China. Zhonghua Yi Xue Za Zhi. 2020;100(14):1106‐1111. 32294877 10.3760/cma.j.cn112137-20191117-02497

[4] Wang G , Wang F , Gibson PG , et al. Severe and uncontrolled asthma in China: a cross‐sectional survey from the Australasian Severe Asthma Network. J Thorac Dis. 2017;9(5):1333‐1344. 28616286 10.21037/jtd.2017.04.74 PMC5465130

[5] Chung KF , Wenzel SE , Brozek JL , et al. International ERS/ATS guidelines on definition, evaluation and treatment of severe asthma. Eur Respir J. 2014;43(2):343‐373. 24337046 10.1183/09031936.00202013

[6] Pavlidis S , Takahashi K , Ng Kee Kwong F , et al. “T2‐high” in severe asthma related to blood eosinophil, exhaled nitric oxide and serum periostin. Eur Respir J. 2019;53(1). 10.1183/13993003.00938-2018 30578390

[7] Moore WC , Bleecker ER , Curran‐Everett D , et al. Characterization of the severe asthma phenotype by the National Heart, Lung, and Blood Institute's Severe Asthma Research Program. J Allergy Clin Immunol. 2007;119(2):405‐413. 17291857 10.1016/j.jaci.2006.11.639 PMC2837934

[8] Shaw DE , Sousa AR , Fowler SJ , et al. Clinical and inflammatory characteristics of the European U‐BIOPRED adult severe asthma cohort. Eur Respir J. 2015;46(5):1308‐1321. 26357963 10.1183/13993003.00779-2015

[9] Kupczyk M , Dahlen B , Sterk PJ , et al. Stability of phenotypes defined by physiological variables and biomarkers in adults with asthma. Allergy. 2014;69(9):1198‐1204. 25039610 10.1111/all.12445

[10] Chung KF . Targeting the interleukin pathway in the treatment of asthma. Lancet. 2015;386(9998):1086‐1096. 26383000 10.1016/S0140-6736(15)00157-9

[11] Kuo CS , Pavlidis S , Loza M , et al. T‐helper cell type 2 (Th2) and non‐Th2 molecular phenotypes of asthma using sputum transcriptomics in U‐BIOPRED A transcriptome‐driven analysis of epithelial brushings and bronchial biopsies to define asthma phenotypes in U‐BIOPRED. Eur Respir J. 2017;49(2):443‐455.

[12] Chung KF , Adcock IM . Precision medicine for the discovery of treatable mechanisms in severe asthma. Allergy. 2019;74(9):1649‐1659. 30865306 10.1111/all.13771

