# Supplementary material for: Severe eosinophilic asthma in Chinese C‐BIOPRED asthma cohort
Source: Clin Transl Med. 2022 Feb 20;12(2):e710. doi: 10.1002/ctm2.710 (PMC8858615; doi:10.1002/ctm2.710)
Supplement: Supplementary file 1 — Supporting Information [file CTM2-12-e710-s001.docx]

**Severe eosinophilic asthma in Chinese C-BIOPRED asthma cohort**

Qingling Zhang ^1, *^, Xiuhua Fu ^2, *^, Changzheng Wang ^3, *^, Huahao Shen ^4, *^, Lei Zhu ^5, *^, Guochao Shi ^6, *^, Zhongmin Qiu ^7, *^, Zhongguang Wen ^8, *^, Wei Gu ^9, *^, Wei Luo ^1, *^, Lina Zhao ^10^, Yunqin Chen ^10^, Sam Lim ^10^, Chang Xiao ^10^, Jian Kang ^12^, Yunhui Zhang ^13^, Mao Huang ^14^, Jinfu Xu ^15^, Kewu Huang ^16^, Qiang Li ^17^, Xiangyan Zhang ^18^, Jianping Zhao ^19^, Xiaoxia Liu ^20^, Shenghua Sun ^21^, Huaping Tang ^22^, Bei He ^23^, Shaoxi Cai ^24^, Ping Chen ^25^, Chunhua Wei ^26^, Guangfa Wang ^27^, Ping Chen ^28^, Lixin Xie ^29^, Jiangtao Lin ^30^, Yuling Tang ^31^, Zhihai Han ^32^, Kian Fan Chung ^11, #^, Nanshan Zhong ^1, #^ on behalf of the C-BIOPRED consortium

**Supplemental materials**

Methods

Figure S1, S2, S3, S4, S5, S6

Table S1, S2, S3, S4

Acknowledgements: C-BIOPRED Consortium

**Methods**

**Participants**

We established a multicentre prospective study that recruited patients with severe asthma from 33 university hospitals in 15 provinces in China between 2015 and 2018. Prior to their enrolment, participants with severe asthma were required to have been under follow-up in a specialised asthma clinic in a University Hospital for at least 6 months, during which time the diagnosis of asthma is confirmed and its severity quantified, with optimisation of asthma control, asthma medications and medication adherence undertaken. The study was approved by the Ethics Committee for each participating institution. All participants gave written and signed informed consent.

**Asthma groups**

Participants were diagnosed with asthma with a history of wheezing, and with demonstration of either airflow reversibility (increase in forced expiratory volume in 1 sec (FEV_1_) >12% of baseline value and >200 mL increase following inhalation of 400 µg of albuterol), or airway hyperresponsiveness (methacholine provocative concentration causing a 20% fall in FEV_1_ <8 mg·mL^−1^). For the classification of severe asthma, the patient should receive treatment with medium-to-high dose ICS and LABA with or without other controller medication, for >6 months prior to baseline visit. Subjects must have uncontrolled asthma and/or have frequent exacerbations (≥2 per year) despite the high level of treatment, or require daily oral corticosteroid (OCS) and/or other controller medication to achieve control.

Four groups of subjects were recruited:

**Group A**: *severe non-smoking asthma (NSA)*

Participants in this group were non-smokers for at least the past 12 months, with a less than 5 pack-year smoking history, with asthma and uncontrolled symptoms defined according to Global Initiative for Asthma (GINA) guidelines (1) and/or frequent exacerbations (more than two per year) despite high-dose inhaled corticosteroids (≥1000 µg fluticasone propionate per day or equivalent dose).

**Group B**: *smokers and ex-smokers with severe asthma (SSA)*

This group was defined as for the NSA group except that they were either current smokers or ex-smokers with a smoking history of at least 5 pack-years.

**Group C**: *mild/moderate non-smoking asthmatics (MMA)*

Participants in this group were non-smokers for at least the past 12 months, with a less than 5 pack-year smoking history and had controlled or partially controlled asthma symptoms, as defined by GINA guidelines (15), whilst receiving a dose of <500 µg fluticasone propionate/day or equivalent.

**Group D**: *healthy non-smoking controls (HC)*

These participants had no history of asthma or wheeze, had no other chronic respiratory disease, were non-smokers for at least the past 12 months with a smoking history of ≤5 pack years and their pre-bronchodilator FEV_1_ was ≥80% of predicted.

**Protocol and assessments**

As shown in **Supplementary** [**Figure S1**](http://erj.ersjournals.com/content/46/5/1308.long#F1)**,** participants attended a screening visit to assess their eligibility for the study. They underwent a baseline visit and were invited to attend for an optional bronchoscopy and high-resolution lung computed tomography*.* At the baseline visit, participants gave no history of any upper or lower respiratory tract infection or symptoms in the previous 3 weeks. Pre- and post-bronchodilator spirometry, haematological profiles, and FeNO were performed. Induced sputum was obtained for measurement of differential sputum eosinophil and neutrophil counts. Allergic status was obtained by measurement of specific immunoglobulin (Ig)E to five common aeroallergens. All participants in the severe asthma cohorts were invited for review at 12-15 months after the baseline visit for a longitudinal visit.

**Pre and post-bronchodilator spirometry**

Spirometry was performed using a portable spirometer calibrated using a 3-litre syringe prior to each study visit. Short-acting β-agonists (SABAs) were withheld for 4 hours and long-acting β-agonists (LABAs) for 12 hours prior to the test. The best values for forced vital capacity (FVC) and forced expiratory volume in 1 second (FEV_1_) were selected from three acceptable manoeuvres. For bronchodilator reversibility, 400μg of salbutamol (4 puffs) was administered by a metered-dose inhaler through a spacer and spirometry was repeated after 15 minutes.

**FeNO measurement**

FeNO was measured using an online single breath analyser (NIOX MINO®, Aerocrine, Stockholm, Sweden) at a flow rate of 50ml/s according to ERS/ATS guidelines (2). The mean of at least 2 measurements was obtained.

**Questionnaires**

The following questionnaires were used: 1) Asthma Control Questionnaire (ACQ5) (3) to assess current asthma control; 2) Asthma Quality of Life Questionnaire (AQLQ) (4) to assess quality of life and psychological morbidity; 3) Epworth Sleepiness Scale (ESS) (5) to measure sleep and daytime drowsiness; and 4) Medication Adherence Report Scale (MARS) (6) to measure adherence.

**Sputum induction and analysis**

Sputum induction was performed using an ultrasonic nebuliser, and inhalation of hypertonic (0.9 to 4.5%) saline using a standardised protocol (7). Participants with a post-bronchodilator FEV_1_ of less than 1.5 L or less that 50% predicted or previous adverse event with hypertonic saline underwent induction starting with 0.9% saline and increasing in increments to 4.5% saline. Sputum plugs were selected and dispersed using 1,4 dithioerythritol. Cell smears were fixed in neutral formalin and stained with hematoxylin-eosin. Differential cell counts were determined by assessment of a maximum of 400 inflammatory cells. Sample viability and a cut-off of <40% squamous cells was the default for samples being made available for analysis.

**Samples collected**

Blood and sputum samples were sent for immediate analysis. Some samples were stored in the local storage facilities before dispatch for storage in a central biobank at AstraZeneca (AZ), Shanghai, until required for analysis.

**Data collection**

An electronic clinical report form (eCRF) using the AstraZeneca electronic database platform was used for the recording of all data which was stored on a secure central database at AstraZeneca, Shanghai. The study was run and monitored by AstraZeneca staff.

**Statistical analysis**

Continuously-distributed data were either summarised using mean± SE if it is baseline characteristics, or median (interquartile range) values for testing results. Discretely distributed data were summarised using frequency and percentage. Missing data were not imputed. P-values were calculated using a Kruskal-Wallis test for continuous variables or Fisher exact test for categorical variables.

**References**

1. Bousquet J. Global initiative for asthma (GINA) and its objectives. Clin Exp Allergy. 2000;30 Suppl 1:2-5.

2. ATS/ERS recommendations for standardized procedures for the online and offline measurement of exhaled lower respiratory nitric oxide and nasal nitric oxide, 2005. Am J Respir Crit Care Med. 2005;171(8):912-30.

3. Juniper EF, Buist AS, Cox FM, Ferrie PJ, King DR. Validation of a standardized version of the Asthma Quality of Life Questionnaire. Chest. 1999;115(5):1265-70.

4. Juniper EF, Guyatt GH, Ferrie PJ, Griffith LE. Measuring quality of life in asthma. Am Rev Respir Dis. 1993;147(4):832-8.

5. Johns MW. A new method for measuring daytime sleepiness: the Epworth sleepiness scale. Sleep. 1991;14(6):540-5.

6. Cohen JL, Mann DM, Wisnivesky JP, Home R, Leventhal H, Musumeci-Szabo TJ, et al. Assessing the validity of self-reported medication adherence among inner-city asthmatic adults: the Medication Adherence Report Scale for Asthma. Ann Allergy Asthma Immunol. 2009;103(4):325-31.

7. Luo W, Chen Q, Chen R, Xie Y, Wang H, Lai K. Reference value of induced sputum cell counts and its relationship with age in healthy adults in Guangzhou, Southern China. The clinical respiratory journal. 2018;12(3):1160-5.

**Supplementary Figure S1** Time-flow and visits of C-BIOPRED study.


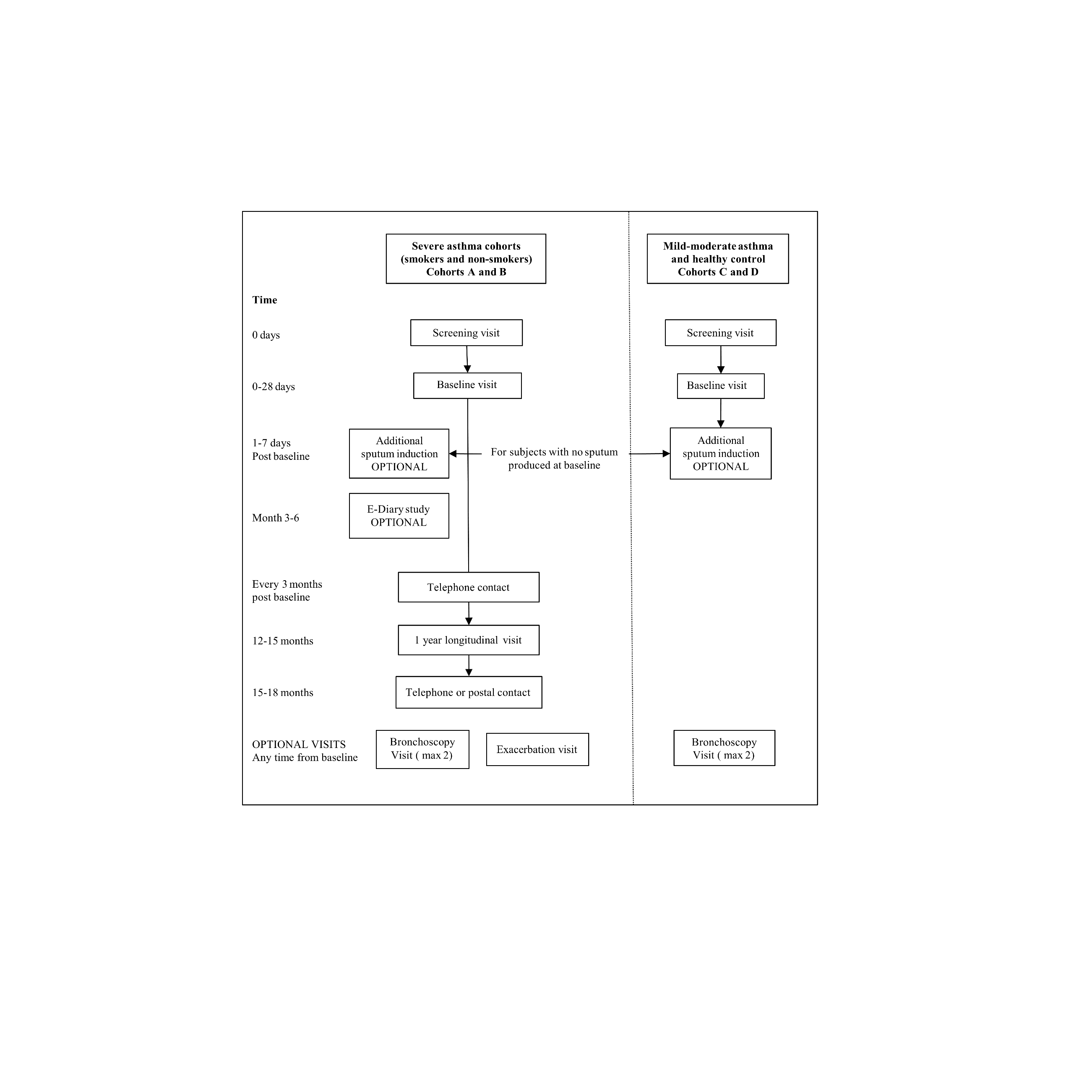


**Supplementary Figure S2** Consort diagram. CT: computed tomography; NA: not applicable.


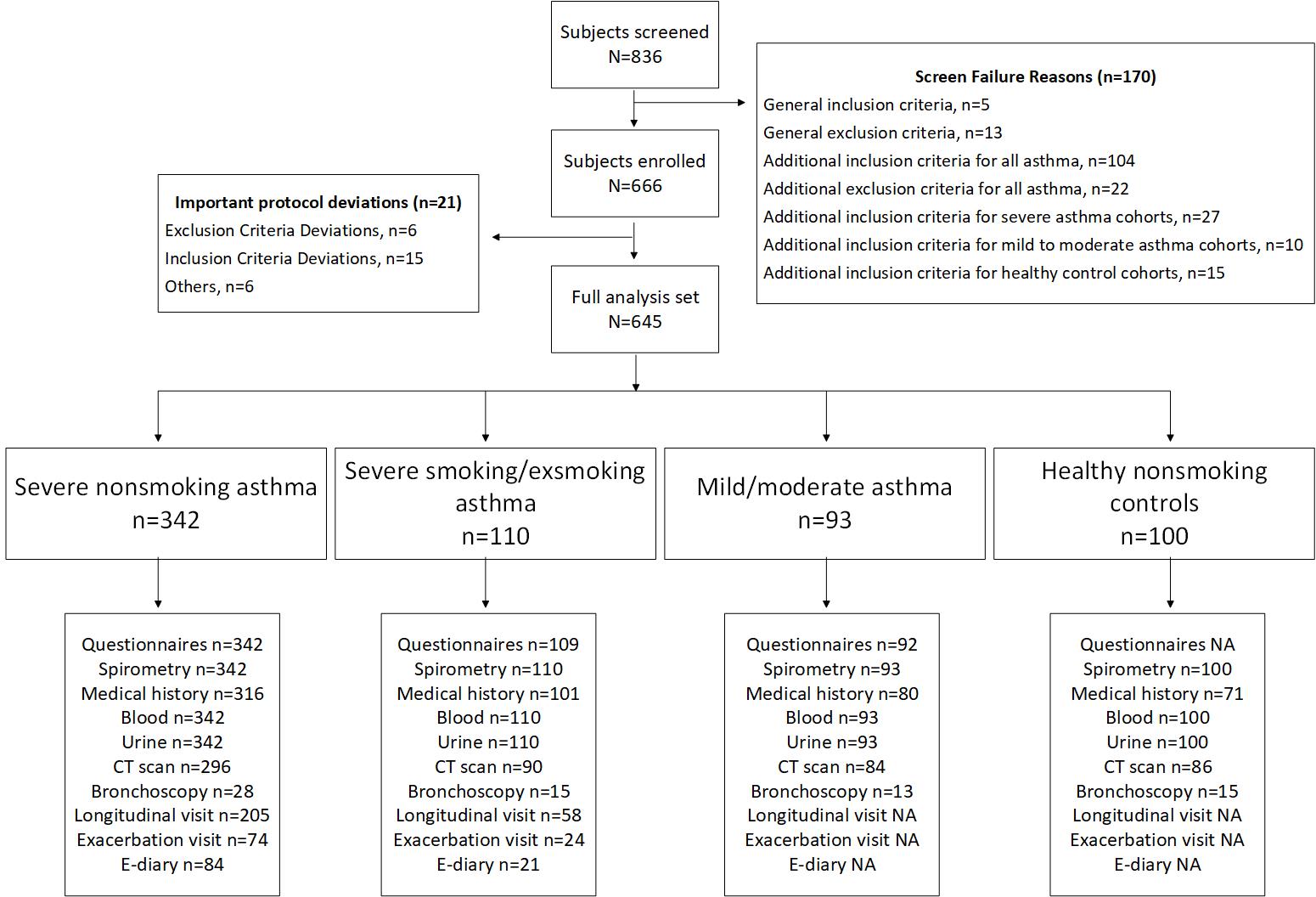


**Supplementary Figure S3. C**orrelations between AQLQ score and FEV1 (% predicted), FEV1/FVC (%), exacerbations in the previous year, ACQ-5 score, BMI and pack-years of smoking for severe asthma and mild-moderate asthma. The correlation coefficients (R) and p-values are indicated.

**
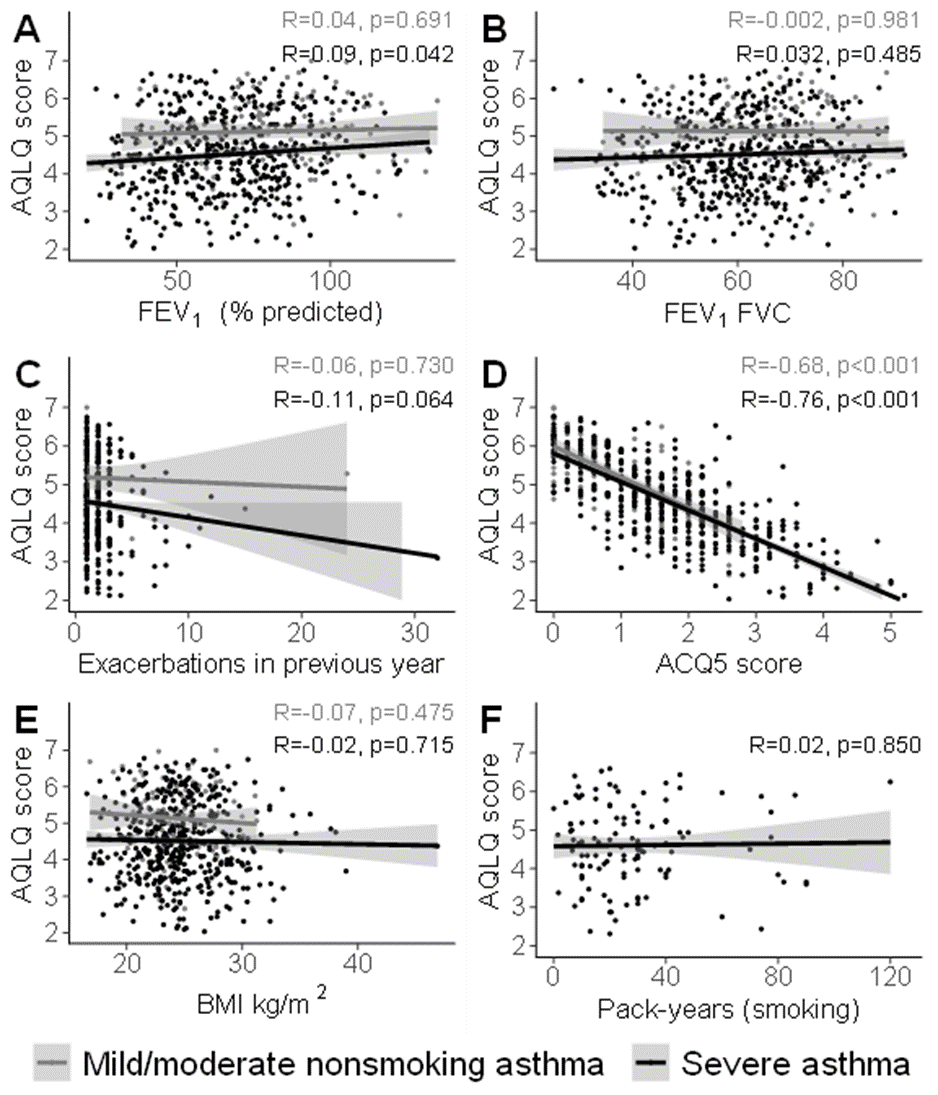
**

**Supplementary Figure S4.** Correlations between blood eosinophils (%) and FeNO, exacerbations in previous year, FEV_1_(% predicted), FEV_1_/FVC ratio, AQLQ score and ACQ-5 score, for severe asthma and mild-moderate asthma. The correlation coefficients (R) and p-values are indicated.


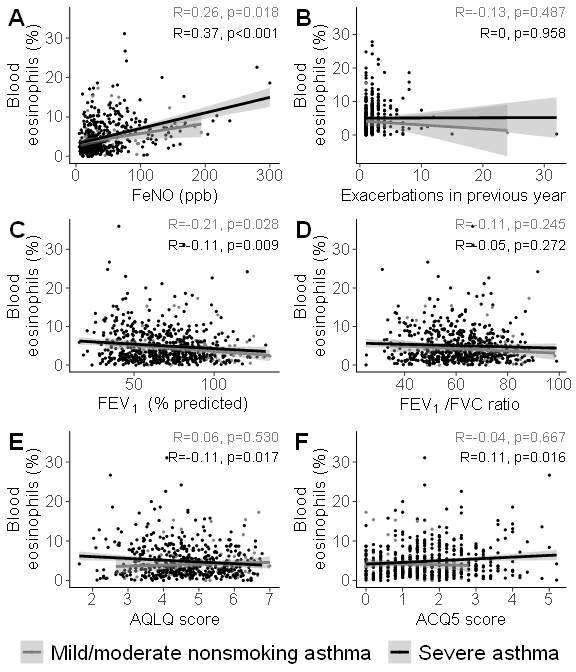


**Supplementary Figure S5.** Sputum eosinophil counts as percent of total cell count by cohort. The boxes represent median and interquartile range values; whiskers extend to 1.5 times of the interquartile range from the box with outliers denoted by the dots. NSA: severe nonsmoking asthma. SSA: smokers and ex-smokers with severe asthma. MMA: mild/moderate nonsmoking asthma. HC: healthy nonsmoking controls.

**
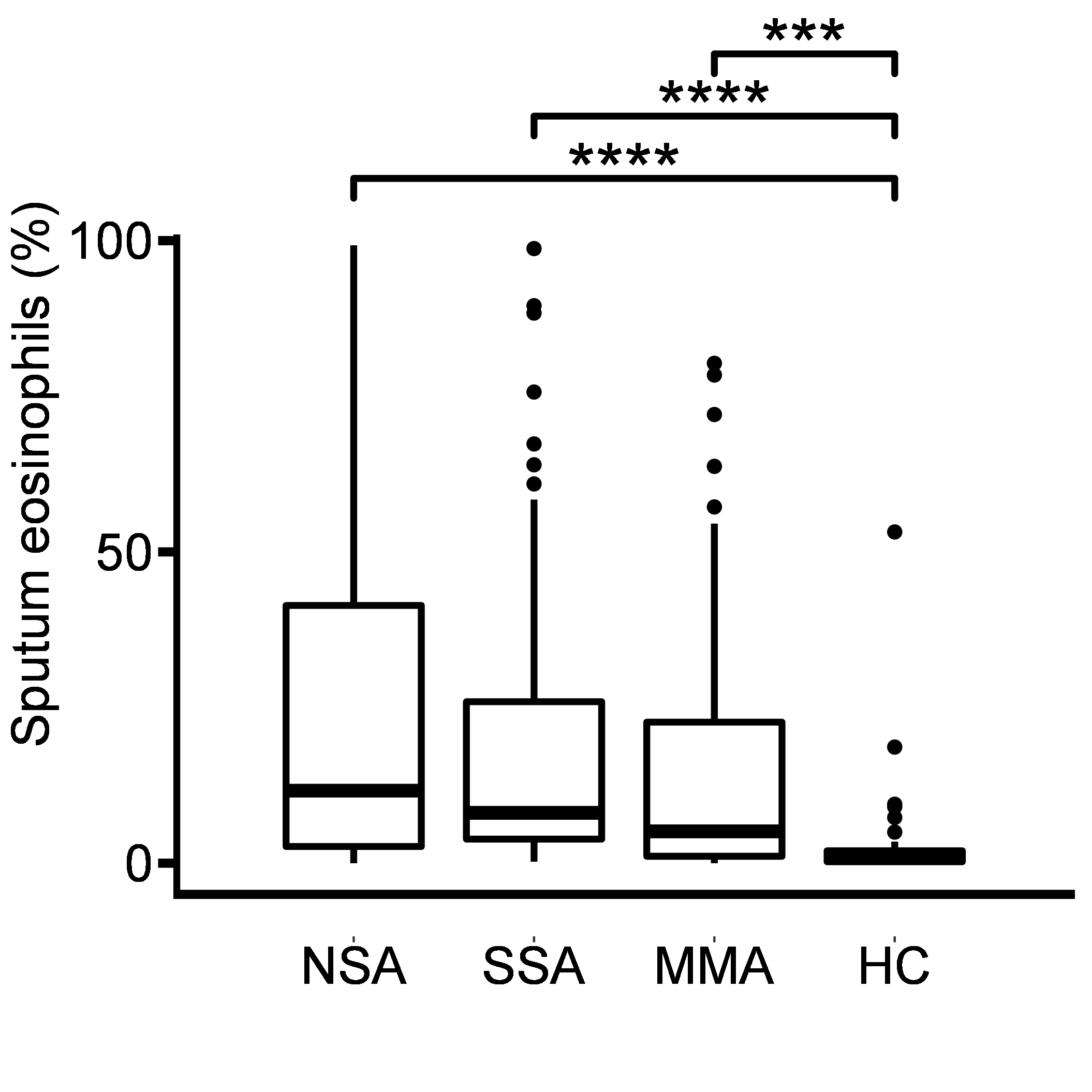
**

**Supplementary Figure S6.** Correlations between sputum eosinophils (%) and blood eosinophils (%), and blood eosinophil counts (A and B, respectively) and between sputum neutrophils (%) and blood neutrophils (%), and blood eosinophils counts (C and D, respectively). The correlation coefficients (R) and p-values are indicated.

**
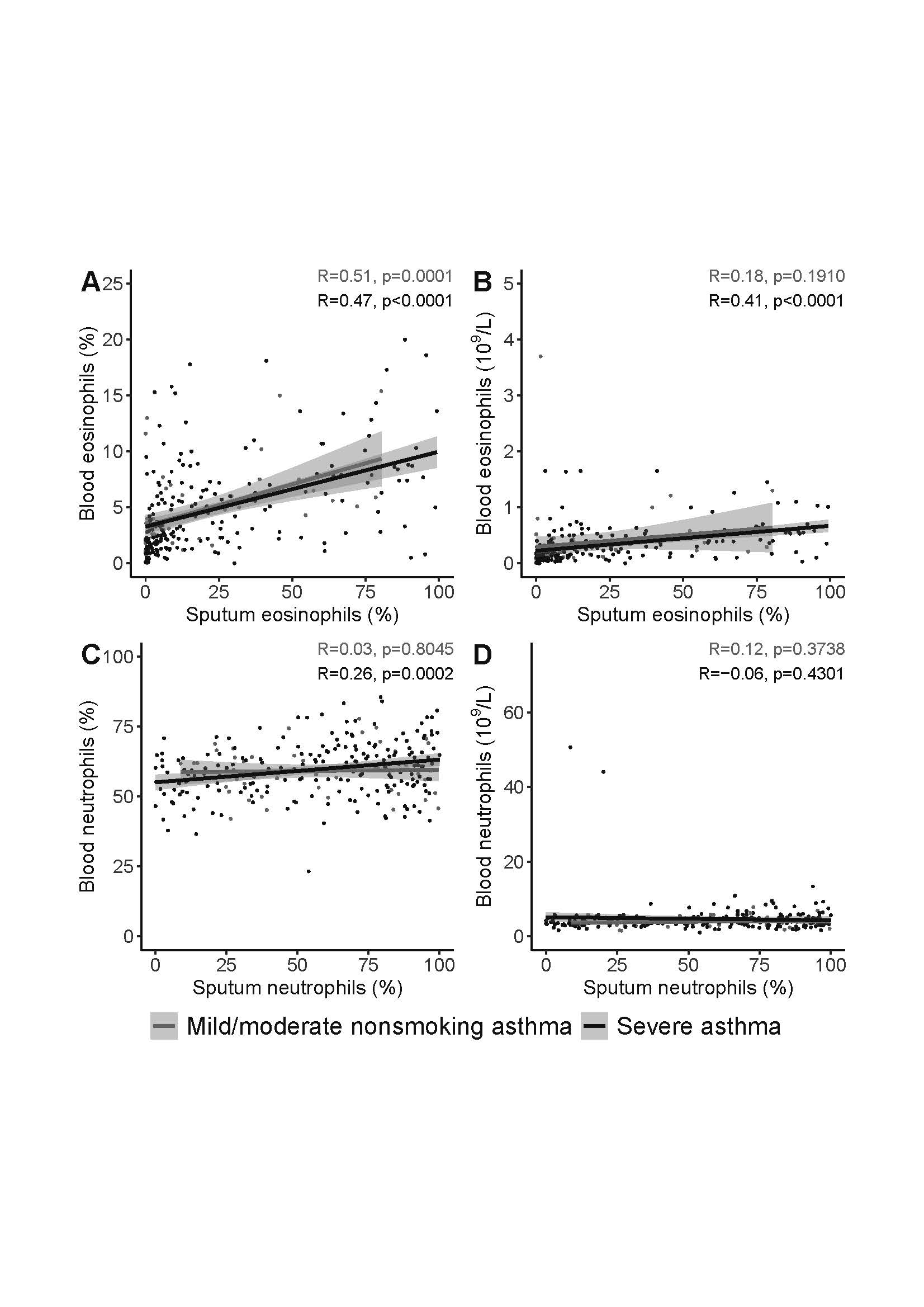
**

| **Supplementary Table S1. Maintenance Medication** | | | |
| --- | --- | --- | --- |
|  | **Number (%) of Subjects** | | |
|  | **Severe nonsmoking asthma (N=342)** | **Smokers and ex-smokers with severe asthma (N=110)** | **Mild/moderate nonsmoking asthma (N=93)** |
| Any asthma medication | 342 (100) | 110 (100) | 93 (100) |
| Antibiotic | 19 (5.56) | 2 (1.82) | 5 (5.38) |
| Antihistamine | 16 (4.68) | 2 (1.82) | 0 |
| Chinese traditional medicine | 45 (13.16) | 15 (13.64) | 6 (6.45) |
| ICS Only | 0 | 0 | 10 (10.75) |
| ICS/LABA | 342 (100) | 109 (99.09) | 82 (88.17) |
| Immunostimulants[a] | 2 (0.58) | 1 (0.91) | 0 |
| Injected glucocorticoids[b] | 5 (1.46) | 3 (2.73) | 0 |
| LAMA | 54 (15.79) | 29 (26.36) | 4 (4.3) |
| Mucolytics | 15 (4.39) | 6 (5.45) | 3 (3.23) |
| Nasal steroid | 1 (0.29) | 0 | 0 |
| OCS[c] | 34 (9.94) | 14 (12.73) | 0 |
| SABA | 47 (13.74) | 14 (12.73) | 7 (7.53) |
| SABA/SAMA | 4 (1.17) | 2 (1.82) | 0 |
| SAMA | 4 (1.17) | 3 (2.73) | 1 (1.08) |
| Singulair/LTRA | 168 (49.12) | 51 (46.36) | 24 (25.81) |
| Xanthines | 58 (16.96) | 19 (17.27) | 2 (2.15) |
| ICS, inhaled corticosteroids; LABA, long-acting beta-agonist; LAMA, long-acting muscarinic antagonist; LTRA, leukotriene receptor antagonist; OCS, oral corticosteroid; SABA, short-acting beta-agonist; SAMA, Short-acting muscarinic receptor antagonist.  [a] Immunostimulants includes the following medications: Bacterial Lysates, Spleen Aminopeptide and Oral Lyophilized Powder.  [b] Subjects who take medication with intravenous for glucocorticoids have been classified as injection glucocorticoids.  [c] Subjects who take medication daily oral glucocorticoids have been classified as OCS. | | | |

| **Supplementary Table S2. Questionnaire scores** | | | | | |
| --- | --- | --- | --- | --- | --- |
|  | **Severe nonsmoking asthma (N=342)** | **Smokers and ex-smokers with severe asthma (N=110)** | **Mild/moderate nonsmoking asthma (N=93)** | **P value** | **P value*** |
| Subjects with questionnaire, n | 342 | 109 | 92 |  |  |
| ACQ |  |  |  |  |  |
| ACQ5 | 1.75 (1.12) | 1.83(1.01) | 1.04 (0.79) | <0.001 | <0.001 |
| ACQ7 | 1.93 (0.92) | 2.04(0.86) | 1.21 (0.69) | <0.001 | <0.001 |
| AQLQ |  |  |  |  |  |
| Total | 4.48 (1.07) | 4.60(1.04) | 5.23 (0.88) | <0.001 | <0.001 |
| Symptoms | 4.71 (1.12) | 4.71(1.06) | 5.44 (0.88) | <0.001 | <0.001 |
| Activity limitation | 4.41 (1.09) | 4.57(1.14) | 5.25 (0.97) | <0.001 | <0.001 |
| Emotional | 4.39 (1.38) | 4.55(1.33) | 5.02 (1.23) | <0.001 | <0.001 |
| Environmental | 4.13 (1.50) | 4.41(1.42) | 4.79 (1.37) | 0.001 | <0.001 |
| ESS | 6.92 (4.35) | 7.62 (4.4) | 6.83 (3.67) | 0.276 | 0.786 |
| MARS | 21.66 (2.85) | 21.68(3.19) | 20.89 (4.22) | 0.678 | 0.450 |
| Data are presented as mean (standard deviation.  ACQ=Asthma Control Questionnaire; AQLQ=Asthma Quality of Life Questionnaire; ESS=Epworth sleepiness scale; MARS=Medication adherence response scale. N=Number of subjects in the cohort. n=Number of subjects included in the analysis. Note: *P value* represents comparison across Severe nonsmoking asthma cohort, Smokers and ex-smokers with severe asthma and Mild/moderate nonsmoking asthma cohort using Kruskal-Wallis test.  *P value*:* Severe vs. Mild/moderate asthma. Kruskal-Wallis test is used. | | | | | |

| **Supplementary Table S3 Comorbidities and Surgical History** | | | | | | |
| --- | --- | --- | --- | --- | --- | --- |
|  | **Number (%) of Subjects** | | | | |  |
|  | **Severe nonsmoking asthma(N=342)** | **Smokers and ex-smokers with severe asthma(N=110)** | **Mild/moderate nonsmoking asthma(N=93)** | **Healthy nonsmoking controls(N=100)** | **P value (Asthma vs. Control)** | **P value (Four**  **Group)** |
| Any comorbidities | 224 (65.5) | 74 (67.3) | 59 (63.4) | 10 (10.0) |  |  |
| Allergic rhinitis | 164 (48.0) | 44 (40.0) | 51 (54.8) | 2 (2.0) | <0.001 | <0.001 |
| Eczema | 51 (14.9) | 27 (24.5) | 17 (18.3) | 5 (5.0) | 0.001 | 0.001 |
| GERD | 48 (14.0) | 19 (17.3) | 12 (12.9) | 1 (1.0) | <0.001 | <0.001 |
| Hay fever | 35 (10.2) | 20 (18.2) | 14 (15.1) | 0 | <0.001 | <0.001 |
| Nasal polyps | 76 (22.2) | 27 (24.5) | 12 (12.9) | 2 (2.0) | <0.001 | <0.001 |
| Rhinitis (not specified) | 37 (10.8) | 17 (15.5) | 4 (4.3) | 2 (2.0) | 0.004 | 0.001 |
| Sinusitis | 27 (7.9) | 10 (9.1) | 3 (3.2) | 1 (1.0) | 0.013 | 0.017 |
| Any surgical history | 49 (14.3) | 14 (12.7) | 8 (8.6) | 2 (2.0) |  |  |
| Nasal polypectomy | 39 (11.4) | 12 (10.9) | 5 (5.4) | 1 (1.0) | 0.001 | 0.002 |
| Sinus operation | 19 (5.6) | 4 (3.6) | 3 (3.2) | 1 (1.0) | 0.103 | 0.235 |
| Note: Each subject may have multiple conditions in this table. Numbers are number of Subjects, % of group in brackets. ‘Asthma’ vs. Control comparison. ‘Asthma’ comprising Severe nonsmoking, smokers and ex-smokers with severe asthma and Mild/moderate nonsmoking. GERD: Gastroesophageal reflux disease. Note: ‘Nasal polyps’ and ‘Sinusitis’ include both surgical and medical history data Note: P value was calculated using Fisher’s exact test. | | | | | | |

| **Supplementary Table S4. Sputum cell analysis** | | | | | |
| --- | --- | --- | --- | --- | --- |
| **Sputum variables** | **Severe nonsmoking asthma** | **Smokers and ex-smokers with severe asthma** | **Mild/moderate nonsmoking asthma** | **Healthy nonsmoking controls** | **p-value¹** |
| n | 152 | 55 | 53 | 34 |  |
| Neutrophils (%) | 61.0 [22.6, 82.5] | 69.1 [37.1, 84.7] | 57.5 [28.6, 83.1] | 62.8 [34.8, 84.9] | 0.6402 |
| Macrophages (%) | 9.1 [ 3.5, 24.7] | 8.5 [ 3.5, 30.4] | 16.5 [ 7.0, 48.1] | 31.4 [10.3, 65.1] | 0.0018 |
| Eosinophils (%) | 11.6 [ 2.7, 41.4] | 8.1 [ 3.9, 25.9] | 5.1 [ 1.1, 22.6] | 0.9 [ 0.2, 2.0] | <0.0001 |
| Lymphocytes (%) | 0.7 [ 0.2, 1.3] | 0.7 [ 0.2, 1.6] | 0.4 [ 0.1, 1.0] | 0.7 [ 0.2, 1.5] | 0.3165 |
|  |  |  |  |  |  |
| Eosinophilic² n (%) | 116 (76.3) | 43 (78.2) | 31 (58.5) | 8 (23.5) | <0.0001 |
| Neutrophilic³ n (%) | 70 (46.1) | 26 (47.3) | 23 (43.4) | 18 (52.9) | 0.8564 |
|  |  |  |  |  |  |
| Eosinophilic Only⁴ n (%) | 74 (48.7) | 28 (50.9) | 21 (39.6) | 3 (8.8) | 0.0001 |
| Neutophilic Only⁴ n (%) | 28 (18.4) | 11 (20.0) | 13 (24.5) | 13 (38.2) | 0.0952 |
| Neutophilic & Eosinophilic⁴ n (%) | 42 (27.6) | 15 (27.3) | 10 (18.9) | 5 (14.7) | 0.3114 |
| Neither of them⁴ n (%) | 8 (5.3) | 1 (1.8) | 9 (17.0) | 13 (38.2) | <0.0001 |
| Continuous data are presented as median [Q1, Q3], Q1=25th percentile, Q2=75th percentile and categorical data as number and percentage. | | | | | |
| ¹ p-values calculated with the Kruskal-Wallis one-way analysis of variance. | | | | | |
| ² Eosinophilic: Eosinophils≥2.5%. | | | | | |
| ³ Neutrophilic: Age 19-39 years, neutrophils≥47.2%, | | | | | |
| Age 40-59 years, neutrophils≥61.9%, | | | | | |
| Age 60-79 years, neutrophils≥75.0%. | | | | | |
| ⁴ Subjects have been stratified into four categories here. | | | | | |

**Acknowledgements：**

**C-BIOPRED Consortium**

**China-Japan Friendship Hospital:** Jiangtao Lin, Yongming Zhang, Nan Su, Xiaoyan Zhang, Qing Zhao, Ying Nong;

**The General Hospital of Shenyang Military:** Ping Chen, Tianyi Zhu, Binbin Ji, Yan Wang, Haitao Zhao, Jinbao Wang, Zhiyuan Zhang, Xiaona Yang;

**Weifang Asthma hospital:** Chunhua Wei, Jing Han, Nong Yu, Qiang Li, Zhifu Yang, Yanhong Liu, Guohua Li, Huadong Guan, Shuping Zhang;

**The Affiliated Hospital of Inner Mongolia Medical University:** Xiuhua Fu, Lihong Wang, Lingxin Meng, Zhenting Sun, Guangqin Cao, Lei Zhao;

**Beijing Chao-Yang Hospital, Capital Medical University:** Kewu Huang, Hong Zhang, Wen Wang, Li An, Yangyu Chen, Yanli Gao;

**Beijing Friendship Hospital, Capital Medical University:** Xiaoxia Liu, Huifen Zhai, Fang Lin, Zhaohui Zhong;

**The First Affiliated Hospital of PLA General Hospital:** Zhongguang Wen, Yan Xiao;

**The First Hospital of China Medical University:** Jian Kang, Linfei Kong, Xinming Su, Xue Yan, Dijia Zhou;

**Qingdao Municipal Hospital:** Huaping Tang, Wei Han, Yi Shu, Tintian Li;

**Chinese PLA General Hospital:** Lixin Xie, Hong Hu, Bo Liu, Yuzhu Li, Longmei Fan, Yue Zhang, Shifeng Zhao;

**Beijing Tongren Hospital, Capital Medical University:** Xiaofang Liu, Yuhong Wang, Xichun Zhang, Xinmao Wang, Pen Bai;

**Peking University First Hospital:** Guangfa Wang, Yan Hu, Zhanwei Hu, Ju Cao;

**Peking University Third Hospital:** Bei He, Rui Wu, Xiaoyan Gai, Hong Zhu;

**Navy General Hospital:** Zhihai Han, Jiyi Meng, Wei Chen, Li Ma, Zhenqian Liu, Chunyang Zhang, Yan Zhang;

**The Second Affiliated hospital of Zhejiang University School of Medicine:** Huahao Shen, Fugui Yan, Bin Shen, Xinghong Wang, Hao Zhang, Yinghua Yin, Jinkai Liu, Yanxiong Mao;

**Jiangsu Province Hospital:** Mao Huang, Xin Yao, Mao Huang, Ji Zhou, Zhenzhen Wu, Ping Yan, Yujie Zhang, Xinling Yang, Ningfei Ji, Fan Fei, Wanzhen Fu, Yi Yang, Yuting Shi, Wangjian Cha;

**Ruijin Hospital Shanghai Jiao Tong University School of Medicine:** Guochao Shi, Min Zhou, Wei Tang, Wei Chen, Jun Zhou, Wenjie Yang;

**Zhongshan Hospital:** Lei Zhu, Meiling Jin, Liping Xue, Li Li, Chun Li, Yin Gong;

**Shanghai General Hospital:** Qiang Li, Guogang Xie, Luhong Bao, Wuping Bao, Nianyun Li, Fengming Ding, Min Zhang;

**Shanghai Tongji Hospital:** Zhongmin Qiu, Li Yu, Jungang Xie, Lingling Yi, Xianghuai Xu, Qiang Chen, Junjun Tang;

**Shanghai Pulmonary Hospital:** Jinfu Xu, Xiaobing Ji, Jingyun Shi, Bo Su, Xiaojun Yang, Jiuwen Bai, Shuo Liang, Kebing Cheng;

**NanJing First Hospital:** Wei Gu, Jiyong Ma, Yun Liu, Jun Ren, Shan Mao;

**The first affiliated hospital of Guangzhou Medical Hospital:** Qingling Zhang, Baoqing Sun, Jiaxing Xie, Rihuang Qiu, Zhiqiang Wang, Peiyan Zhen, Wenting Luo, Changgao Zhong, Wei Luo, Yanqing xie, Xinxin Yu, Wenting Yu, Wei He;

**Xinqiao Hospital, Army Military Medical University:** Changzheng Wang, Qianli Ma, Min Wan, Mingzhou Zhang, Haining Li;

**Nanfang Hospital of Southern Medical University:** Shaoxi Cai, Haijin Zhao, Yanmei Ye, Fang Zhou, Laiyu Liu, Guohua Huang, Jianpeng Liang;

**Tongji Hospital, Tongji Medical College, Huazhong University of Science and Technology:** Jianping Zhao, Jungang Xie, Qiongjie Hu, Kaiyan Li, Wang Ni, Guohua Zheng, Weining Xiong, Lingling Yi, Xihu Du;

**The First people's Hospital of Yunnan Province:** Yunhui Zhang, Zhi Li, Xiaoqiong Tan, Feng Liao, Shenglan Wang;

**Hainan General Hospital:** Yijiang Huang, Xinjun Cai, Kai Liu, Shaojing Sheng, Yihui Fu;

**The Third Xiangya Hospital of Central South University:** Shenghua Sun, Qiang Zhang, Jinweng Cai, Ye Ling;

**The Second Xiangya Hospital of Central South University:** Pin Chen, Hong Luo, Lv Liu, Zhijun Liu;

**The First hospital of Changsha:** Yuling Tang, Yusheng Yan, Guiyan Mo, Long Wen;

**The Third hospital of Changsha:** Yinqun Zhu, Du Fan.
